# Total Anomalous Pulmonary Venous Connection: Post Operative Problems and Management

**Published:** 2009-02

**Authors:** Sandeep Khanna, Minati Choudhury, Usha Kiran

**Affiliations:** Senior Resident, Department of Cardiac Anaesthesia, Cardiothoracic Centre, All India Institute of Medical Sciences; Associate Professor, Department of Cardiac Anaesthesia, Cardiothoracic Centre, All India Institute of Medical Sciences; Professor and HOD, Department of Cardiac Anaesthesia, Cardiothoracic Centre, All India Institute of Medical Sciences

**Keywords:** Total anomalous pulmonary venous connection, Clinical outcome

## Abstract

**Summary:**

The uncommon congenital cardiac anomaly, total anomalous pulmonary venous connection is incompatible with life unless early surgical intervention is done. Most of the post operative problems and mortality in these children are also due to the changes in pulmonary vasculature. We hereby report two such cases that experienced a stormy postoperative course.

## Introduction

Total anomalous pulmonary venous connection (TAPVC) is a very uncommon cyanotic anomaly comprising 1% of all congenital heart diseases. Since pulmonary veins drain into the systemic venous circulation, TAPVC is incompatible with life unless a communication between the right and left sides of the heart exists; usually via a patent foramen ovale or atrial septal defect.^1^ As the right to left shunt is usually small, right heart dilatation and failure ensues owing to a volume overload. Stenosis and obstruction of varying degree at the junction of the anomalous trunk with the vena cava leads to severe pulmonary hypertension which further worsens right heart failure. Patients present in early infancy with bluish discoloration exaggerated by activity and symptoms of heart failure. They are usually severely acidotic and cyanotic. Without surgery most infants die by 12 months of age. However, post-operative mortality is also high owing to increased pulmonary vascular resistance and indequate repair due to obscure anatomy.^2^ The purpose of this case report is to highlight the fact that best postoperative care is the key element of thankful outcome. Even with best of care misdiagnosis can happen.

## Case 1

A 7-month-male child weighing 4.2kg, presented with complaints of rapid breathing and bluish discoloration of face for 5 days, was diagnosed to have supracardiac TAPVC and a large atrial septal defect (ASD) with right to left shunt. Patient was posted for a reparative surgery on an elective basis. Although no premedication was administered, parental separation was peaceful. The operating room temperature was maintained at 20 degree Celsius. A warming blanket underneath and an overhead warming light were used to keep the patient warm until induction. The induction technique and monitoring during cardiopulmonary by-pass was similar to any other high risk infant who has to undergo open heart surgery. Balanced anaesthesia was maintained with 50% oxygen in air, incremental doses of fentanyl, midazolam and pancuronium bromide. Tidal volume and respiratory rate were adjusted to maintain carbon dioxide homeostasis(Table 1) Anti-coagulation was achieved with heparin 4 mg.kg^−1^ and activated clotting time (ACT) was determined to be greater than 480 seconds at the time of placement of aortic purse string sutures. During cardiopulmonary bypass systemic hypothermia to 28 degree Celsius and cold blood cardioplegia were employed. A pulmonary artery pressure (PAP) monitoring line was placed by the surgeon on bypass. Cardiopulmonary bypass time was 116 minutes and aortic cross clamp time was 80 minutes. Patient was successfully weaned off bypass on dopamine (5 mcg.kg^−1^.min^−1^), nitroglycerine (NTG(1 mcg.kg^−1^.min^−1^), milrinone (0.1 mcg.kg^−1^.min^−1^) as continuous infusion. Post-bypass vital parameters were invasive blood pressure (IBP):70/50mmHg, right arterial pressure (RAP):5mmHg, pulmonary artery pressure (PAP):19/12 mmHg and SaO_2_:100%. Patient was shifted to the intensive care unit where he immediately experienced an episode of pulmonary hypertensive crisis. Vital parameters were as follows: IBP:30/20mmHg, PAP: 70/48 mmHg and SaO_2_: 60%. The baby also had bleeding from endotracheal tube. Ventricular fibrillation ensued; ACLS (advanced cardiac life support) protocol was initiated and the patient was successfully resuscitated after 30 minutes. He was mechanically hyperventilated and received nitric-oxide (NO) 40ppm to decrease pulmonary artery pressures (PAP). One hour later, his vital parameters were – IBP: 60/40 mmHg, PAP:55/35 mmHg and SaO_2_:88%. Tapering of NO therapy was started from 5^th^ post operative day (POD) and was stopped on 12^th^ POD (PAP: 29/15 mmHg). Patient was tracheostomized on 10^th^ POD and was successfully weaned off the ventilatoron 17^th^ POD. Tracheostomy was closed on 20^th^ POD and the patient was shifted to the ward thereafter, from where he was discharged home.

**Table 1:** Ventilation parameters at different time period

	Time (Pre CPB)			Time (Post CPB)			
	PI	PI	PI	PC	PC	PC	PC
	0min	15min	30min	0min	15min	30min	45min
**Case-1**							
***(4.2 kg)***							
VT	45	55	55	45	55	55	55
*RR(min)*	35	30	30	40	35	35	35
*PaCO*_2_*mmHg*	46	40	-	54	40		35
*EtCO*_2_*mmHg*	66	54	42	55	42	-	32
**Case-2**							
***(2.8 kg)***							
*VT*	30	35	35	30	30	30	30
*RR(min)*	40	40	40	40	40	40	40
*PaCO*_2_	48	40	-	36	42	-	36
*mmHg*							
EtCO_2_	70	50	38	38	38	35	30
*mmHg*							

PI: post intubation, PC: post cardiopulmonary bypass, VT: tidal volume, RR: respiratory rate, EtCO_2_:end tidal carbon dioxide, PaCO_2_:partial pressure of carbon dioxide

## Case 2

A 5-month-male child weighing 2.8kg was diagnosed to have supracardiac TAPVC and unrestrictive ASD. Anaesthetic management was similar to case 1. Patient was successfully weaned off bypass with (dobutamine) and vasodilator therapy (NTG). Post-operatively patient was mechanically hyperventilated to keep the PAP low. However in view of increasing requirement of inotropes, high RAP and non responsive to NO therapy pressures, the adequacy of repair was questioned. Cardiac catheterization and angiography revealed that the right pulmonary vein was still draining into the azygous vein. Patient was taken up for surgery again on 3^rd^ POD to re-route the anomalous vein to the LA but since it was received that the patient would not be able to tolerate another stresson cardiopulmonary bypass, the azygous vein was ligated instead. On 8^th^ POD, patient was tracheostomized. Soon after he developed an episode of bradycardia, followed by asystole and could not be resuscitated.

## Discussion

In TAPVC, the pulmonary vasculature often has a thickened medial layer; thus, PVR does not decrease normally after repair and the right ventricle has to pump against an increased after load. The left ventricle is unable to support the circulation probably because it is under filled and underutilized prior to correction. Its function is also hampered by septal displacement. Thus, the postoperative period is complicated by existence of a low output state, persistence of pulmonary hypertension (PHT) and a highly reactive pulmonary vasculature^1^. The abnormal pulmonary vasculature is highly susceptible to change in blood gases and lung mechanics. Morray et al demonstrated a 50% increase in PAP when the PaCO_2_ was increased from less than 30mmHg to 40-45mmHg in the post-operative period in children with reactive pulmonary vasculatures who underwent corrective cardiac surgery. Thus mechanical ventilation to a PaCO_2_ to 30-35 mmHg is mandatory in the postoperative period. In addition, by stretching the lungs, positive pressure ventilation leads to release of prostaglandins which cause pulmonary vasodilation^2^ PAP can also be pharmacologically altered. Most available drugs act both on pulmonary as well as systemic vasculatures and hence their utility is limited by their propensity to decrease blood pressure and coronary flow to the ventricles. However, in recent years inhaled NO has emerged as a relatively specific pulmonary vasodilator. It rapidly diffuses through the alveolar capillary membrane and activates soluble guanylate cyclase leading to smooth muscle relaxation. Since it acts directly on vascular smooth muscle, it remains effective in reducing PVR despite the post CPB endothelial injury encountered frequently in children. Its efficacy in the management of PHT has been repeatedly demonstrated and it has been hailed as the wonder drug of decade^3^–^6^. Typically, doses used have varied from 5-80ppm^3^–^6^ Advantages include rapid inactivation by hemoglobin, no systemic hypotension and consistent effect in decreasing PAP. However, NO is an expensive drug. In addition costs are increased by the need for a specialized delivery system and requirement for waste gas scavenging. Other potential adverse effects include methemoglobinemia and nitrogen dioxide toxicity, rebound pulmonary hypertension after withdrawal and pulmonary vascular congestion in patients with poor left ventricular function. These side effects are rare if NO is used in prescribed clinical doses.^3^–^6^

The incidence of pulmonary hypertensive (PHT) crisis may be as high as 40% in the postoperative period and is a major cause of mortality^7^. Thus its prevention and management are imperative for a successful postoperative outcome.^7^–^8^ PHT crisis is defined as PAP more than or equal to systemic arterial pressures along with significant deterioration in hemodynamic status^8^–^9^ In the absence of direct PAP measurements, it can be suspected in event of unexplained tachycardia, high central venous pressure (CVP), hypotension and desaturation. Once detected, it can be managed in the following manner:

**Table:** Management of pulmonary artery hypertensive crisis

Treatment	Rationale
Administer 100%oxygen	Increasing PaO_2_ can decrease PVR
Hyperventilation	A decrease in PaCO_2_ leads to decrease in PAP
Chest X-rayto rule out pneumothorax	Pneumothorax causing a decrease in PaO_2_ and to increase in PAP
Correction of metabolic acidosis	PVR has a direct relation to H+ ion concentration
Administration of pulmonary vasodilator	To decrease the PAP
Support cardiac output	Adequate preload and inotropic support
Attenuate pain	Pain and other noxious stimuli can increase PAP

PaO_2_: Partial pressure of oxygen, PAP: Pulmonary artery pressure, PVR: Pulmonary vascular resistance.

In case 1, continuous PAP and IBP measurements allowed us to make a rapid diagnosis of PHT crisis and it was managed in accordance with the principles outlined above. NO, commenced initially at a dose of 40 ppm was tapered off over a period of 10 days and there was no rebound pulmonary hypertension. We used NO in the recommended dose range and encountered no adverse effects. In case 2, since PAP was not monitored, PHT was assumed on the basis of a high CVP, unexplained tachycardia, hypotension and desaturation. Similar management principles were applied but were not successful hence we assumed that the repair was inadequate. Kovalchin and Beghetti in their study demonstrated that, as a selective pulmonary vasodilator, inhaled nitric oxide is an important agent to differentiate pulmonary vasoconstriction from fixed anatomic obstruction or secondary to pulmonary vasospasm.^10^–^11^

To conclude, successful outcome in TAPVC calls for ameticulous preoperative work up and postoperative prevention and management of PHT. As a selective vasodilator, NO acts as an important diagnostic and therapeutic agent in the management of children with TAPVC and other congenital heart disease associated with PAH. Though no randomized placebo controlled trial has been published; several reviews and our own experience purport that except the haemodynamic benefits in PAH children, nitric oxide can also be used to help discriminate anatomic obstruction to pulmonary blood flow from pulmonary vaso-constriction.

## References

[1] Raisher BD, Grant JW, Martin TC (1992). Complete repair of TAPVC in infancy. J Thoracic Cardiovasc Surg.

[2] Morray JP, Lynn AM, Mansfied PB (1998). Effect of pH and PaCO_2_ on pulmonary and systemic hemodyanamics after surgery in children with congenital heart disease and pulmonary hypertension. J Pediatr.

[3] Journis D, Pouard P, Mouriat P (1994). Inhaled NO as a therapy for pulmonary hypertension after operation for congenital heart defects. J Thorac Cardiovasc Surg.

[4] Beghetti M, Habre W, Friedelli B (1995). Continuous low dose inhaled NO for treatment of severe pulmonary hypertension after cardiac surgery in paediatric patients. Br Heart J.

[5] Paggell IAM, Zwass MS, Fineman JR (1998). The effect of inhaled NO on postoperative pulmonary hypertension in infants and children undergoing surgical repair of congenital heart disease. Anesth Analg.

[6] Konstadt S (1995). Nitric oxide: has it progressed from molecular year to wonder drug of decade?. J Thorac Cardiovasc Surg.

[7] Bando K, Tumentine MW, Sharp TG (1996). Pulmonary hypertension after operation for congenital heart disease: analysis of risk factors and management. J Thorac Cardiovasc Surg.

[8] Emdberg L, Olsson AK, Jogi P (2002). How common is pulmonary hypertension after pediatric cardiac surgery?. J Thorac Cardiovasc Surg.

[9] Ruscell IA, Zwass MS, Fineman JR, Balea M, Rouine – Rapp K, Brook M (1998). The effects of inhaled nitric oxide on post operative pulmonary hypertension in infants and children undergoing surgical repair of congenital heart disease. Anesth Analg.

[10] Kovalchin JP, Mott AR, Rosan KL, Feltes TF (1997). Nitric oxide for the evaluation and treatment of pulmonary hypertension in congenital heart disease. Tex Heart Inst J.

[11] Beghetti M, Morris K, Cox P, Bohn D, Adatia I (1999). Inhaled nitric oxide differentiates pulmonary vasospasm from vascular obstruction after surgery for congenital heart disease. Intensive Care Med.

